# Patient satisfaction with quality of care at out-patient departments in selected health facilities in Kumasi, Ghana

**DOI:** 10.1186/s12913-024-11399-w

**Published:** 2024-09-04

**Authors:** Abigail Anima Owusu, Kingsley Boakye, Daniel Boateng, Christa Osei-Mensah, Peter Agyei-Baffour

**Affiliations:** 1 Department of Health Policy, Management, and Economics, School of Public Health, Kwame Nkrumah University of Science and Technology, Kumasi, Ghana; 2 Department of Epidemiology and Biostatistics, School of Public Health, Kwame Nkrumah University of Science and Technology, Kumasi, Ghana; 3 Department of Global Public Health and Bioethics, Julius Center for Health Sciences and Primary Care, University Medical Center, Utrecht University, Utrecht, The Netherlands; 4 Department of Physician Assistantship and Allied Sciences, Garden City University College, Kenyase, Kumasi Ghana

**Keywords:** Public perception, Patient satisfaction, Out-patient department, Quality care, Kumasi

## Abstract

**Background:**

Health care is an indispensable element for economic growth and development of individuals and nations. Healthcare service quality is associated with patient satisfaction, ensuring the safety and security of patients, reducing mortality and morbidity, and improving the quality of life. Patient satisfaction with health service is linked to increased utilization following contendness with healthcare received from health providers. There is an increasing public perception of poor quality of care among patients visiting public health facilities in Ghana which translates into service dissatisfaction. Meanwhile, patient dissatisfaction will more likely result in poor utilization, disregard for medical advice, and treatment non-adherence. The study was conducted to assess patients’ satisfaction with quality of care at the outpatient departments of selected health facilities in Kumasi, Ghana.

**Methods:**

An institutional-based analytical cross-sectional study was conducted among patients (aged ≥ 18 years) visiting outpatient departments of selected health facilities in Kumasi from October - December, 2019. A systematic sampling technique was adopted to collect quantitative information from 385 respondents using a structured questionnaire. At 95% confidence interval and 5% alpha level, two-level logistic regression models were performed. Model I estimated the crude associations and the effect of covariates was accounted for in Model II. The results were presented in odds ratio with a corresponding 95% confidence interval. All analysis were performed using STATA statistical software version 16.0.

**Results:**

Out of the 385 participants, 90.9% of the participants were satisfied with the services they received. Being married [AOR = 3.06, 95%CI = 1.07–8.74], agreeing that the facility is disability-friendly [AOR = 7.93, 95%CI = 2.07–14.43], facility has directional signs for navigation [AOR = 3.12, 95%=1.92–10.59] and the facility has comfortable and attractive waiting area [AOR = 10.02, 95%CI = 2.35–22.63] were associated with satisfaction with health service among patients. Spending more than 2 h at the health facility [AOR = 0.45, 95%CI = 0.04–0.93] and having perceived rude and irritating provider [AOR = 0.14, 95%CI = 0.04–0.51] had lower odds of satisfaction with health service received.

**Conclusion:**

There is a high patient satisfaction with services received at out-patient departments which is influenced by a multiplicity of factors; being married, and agreeing that the facility is disability-friendly, has directional signs for navigation, and the waiting area is comfortable and attractive. The study findings call for the need to develop and implement health delivery interventions and strategies (i.e. patient-centered interventions, disability-friendly facilities, and sustainability and improvement of quality service) to improve and sustain patient satisfaction levels with health care service. These strategies must be directed towards addressing inequalities in infrastructural development and inputs needed for healthcare delivery in the health system.

**Supplementary Information:**

The online version contains supplementary material available at 10.1186/s12913-024-11399-w.

## Introduction

Healthcare is an indispensable element for economic growth and development of individuals and nations [1]. Healthcare service quality is associated with patient satisfaction [2], ensuring safety and security, reducing mortality and morbidity, and improving quality of life patients. Within developed countries, high quality of health care delivery is mostly associated with the healthcare system but differs in developing countries like Ghana. For many low and middle-income countries (LMICs), poor quality health care is now responsible for a greater number of mortalities than insufficient access to care [3]. Since healthcare organizations are operating in an increasingly competitive and dynamic environment, patient satisfaction is crucial and central to healthcare delivery, yet patients’ satisfaction are rarely considered by providers [4]. Realising the universal health coverage target of ‘all people having access to health services’, means that all people must be satisfied in order to continuously access healthcare. Thus, understanding patients’ experience and satisfaction with health services delivery is important to monitor and improve the quality of care [5], for high utilization.

Patient’s satisfaction is a relative phenomenon that embodies the patients’ perceived need, expectations, and experience of health care [6]. Healthcare quality is perceived as poor in developing countries, although this is partly attributed to being influenced by better quality healthcare exhibited in developed countries [5]. Outpatient departments (OPDs) are the first points of contact of health facilities with patients, serving as the shop window to any healthcare service provided to the community. Therefore, the care received at the OPD mirrors the quality of services of the health facility and is reflected by patients’ satisfaction with the services being provided [7].

In most developing countries, evidence has revealed patients’ satisfaction has been remarkably low. Health facilities have to be patient-centred and focus on service marketing to ensure the sustainability of their services [8]. In Ghana, the Ministry of Health (MoH) oversees the formulation of policies and strategies, and healthcare for the populace are generally implemented by the implementing partners of MoH (Ghana Health Service, Christian Health Association of Ghana, Mission-based and private facilities, among others). The MoH coordinates the implementation of primary health care policy with other ministerial agencies [9]. The two major cities and regions in Ghana (Greater Accra and Ashanti regions) have typically attracted the best health facilities and significant proportion of health staff [10], and these professionals provide health services to patients, including out-patient services.

The out-patient health service simply denotes the provision of health service to patients at health facilities where they are managed and discharged the same day [11]. Some of these out-patient services receive by patient encompass general health services such as triage, health promotion and preventive services and general medical surgical, pediatric and maternal services [11], offering these line of treatments of primary care in a safe and pleasant environment. Studies in Ghana have revealed a fierce competition between public and private health providers, as healthcare quality has become a critical component of national healthcare policies [8, 12]. Patients seem to prefer private health care facilities despite the fact that public health providers have a more complex structure and employ the bulk of health experts in the country [8, 12].

In some instances, patients at public facilities are misdiagnosed and poorly treated, often forcing them to resort to treatment at private facilities [13]. Perceived effectiveness, confidence, service quality, interpersonal relationship, and shorter waiting time influence patients’ preference of private health facilities [14]. Additionally, physical environment and outcome quality influence choice of facilities patients visit [13]. Despite the perceived benefits and quality associated with utilizing private health facilities to public, majority of Ghanaians seem to rely often on public health facilities for healthcare services. The plausible justification could be due to the higher cost of accessing private healthcare facilities, and poor financial status among many Ghanaians. This percentage of Ghanaians utilizing public health facilities experience sub-optimal quality care, translating into poor patient satisfaction [14].

There is an increasing perception of poor quality healthcare among public health facilities in Ghana. Although, several efforts have been made by the Government of Ghana to improve healthcare quality and services in the country. Meanwhile, it has become increasingly clear that this perceived poor quality of care results in patients’ dissatisfaction, as well as acts as affront to healthcare utilization [15]. Quality of care is a major factor that could explain the disparities and the unsatisfactory health outcomes [16]. Patients who are dissatisfied with health services are less likely to utilise health facility, disregard medical advice, and not adhere to treatment regimen [17].

Even though, some studies have been conducted on patient satisfaction with health services in Ghana, the authors limited the studies to Greater Accra and Volta regions [18–20], and Bono region [21]. As a result, there is still dearth of literature on the satisfaction levels of patients on health services in Kumasi, Ashanti region where most health facilities in Ghana are concentrated. This study extends the previous literature, and contributes to bridging the research gap by conducting a cross-sectional study on patient satisfaction with quality of care at the out-patient departments in selected health facilities in Kumasi.

## Methods

### Study design and setting

An institutional-based analytical cross-sectional study was conducted among patients from October – December, 2019 to assess their satisfaction with health services received at various OPDs in three selected health facilities in Kumasi, Ghana.

**Fig. 1:**
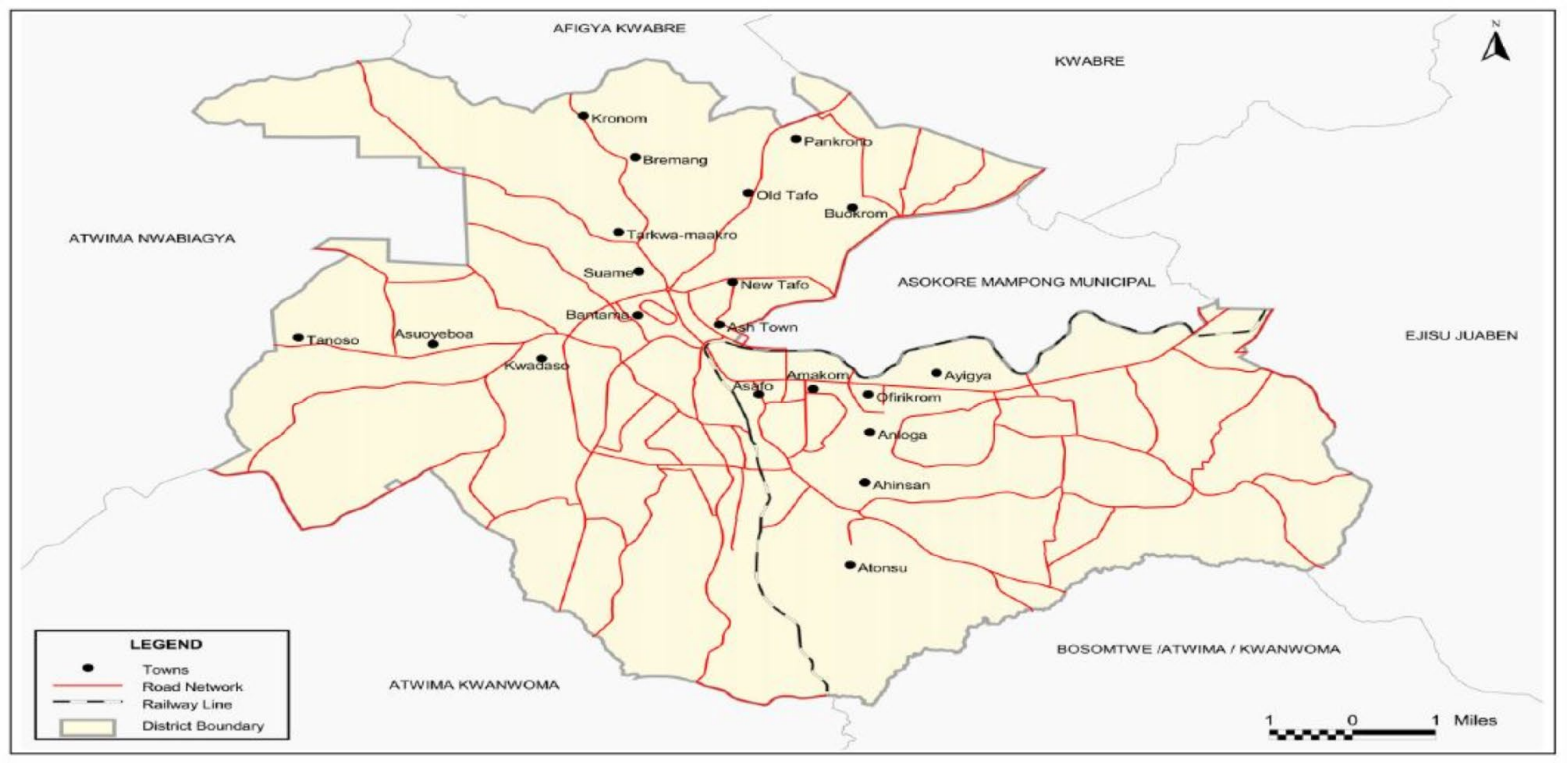
Map of Kumasi metropolis. *Source*: Ghana Statistical Service, (2010)

The Kumasi Metropolitan is one of the 42 administrative districts in the Ashanti Region of Ghana with Kumasi as the district capital [22]. It is 250 to 300 m in altitude and lies between Latitude 6.35^o^N and 6.40^o^S and Longitude 1.30^o^W and 1.35^o^E. The Kumasi Metropolitan Assembly (KMA) was created by Legislative Instrument 1614 of 1995, which amended the Local Government Act 462, 1993, with the Local Government Legislation 1988, NDPC law 207. It houses about 36.2% of the region’s populace. According to the 2021 population and housing census, Kumasi Metropolitan has a population of 443,981 with 213,662 males and 230,319 females [22]. Currently, the Kumasi Metropolis has about 50 health facilities (including both public, Christian Health/Mission-based facilities, and private institutions) at different levels (primary, secondary, and tertiary) providing healthcare services to its residents. Among the existing health facilities within the metropolis, inhabitants frequently visit public health facilities for healthcare. Three facilities (Kumasi South Hospital, Cocoa Clinic, and Tafo Hospital) were selected out of 50 health facilities in the Metropolis for the study. These facilities selected for the study are public health facilities and they provide client’s walk-in services to patients. The facilities provide both primary and secondary level care to patients that visit these facilities. These facilities were selected because they are among the health facilities residents in Kumasi mostly visit for health care services. Additionally, they were selected because they are the immediate referral facilities within the district they are located, therefore recording high outpatient attendance monthly (See Fig. 1).

### Study population

The study population were patients who visited the various OPDs in the three selected health facilities in Kumasi (i.e. Kumasi South Hospital, Cocoa Clinic, and Tafo Hospital).

### Inclusion criteria

The study included adult patients (≥ 18 years) who visited the three selected health facilities for outpatient services, not critically ill, and were of sound mind to make objective judgments.

#### Exclusion criteria

Adult patients who met the inclusion criteria, but were critically ill, were admitted even though visited the facility for out-patient services, and those mentally challenged to make objective judgments were excluded from the study.

#### Sample size calculation

The sample size estimated for the study was 385 patients, computed following the formula for calculating sample size for cross-sectional studies by Charan and Biswas in 2013 [23]. The researchers assumed that the level of patient satisfaction was similar to an investigation conducted by Shure and colleagues [24].

n = $$\:\frac{{Z}^{2}\:pq}{{e}^{2}}$$

Where n is the sample size.

Z^2^ is the desired confidence level, e.g., 95%, which is constant at 1.96.

e is the desired level of precision at a confidence level of 95%.

p is the estimated proportion of patient satisfaction from previous study.

Therefore,

n = $$\:\frac{{\left(1.96\right)}^{2}\:\left(0.65\right)\left(0.35\right)}{{\left(0.05\right)}^{2}}$$

*n* = 350.

However, 10% was added to cater for non-response (10% of 350 = 35).

Hence, the estimated sample size for the study was 385.

The total number of clients sampled per facility was shared proportionally across the three facilities based on the average daily OPD attendance (probability proportional to size) (see Table 1).

**Table 1:** Distribution of respondents among health facilities

Health Facility	Average attendance for June 2019	% Attendance	Number of Participants
Kumasi South Hospital	5175	46	177
Cocoa Clinic Kumasi	2320	21	81
Tafo Government Hospital	3638	33	127
**Total**	**11**,**133**	**100**	**385**

### Sampling strategy

A systematic sampling technique was adopted to assess the satisfaction of patients on quality of service received at OPDs in these selected facilities. These facilities were selected because they are among the health facilities residents in Kumasi mostly visit for health care services. Additionally, they were selected because they are the immediate referral facilities within the district they are located, therefore recording high outpatient attendance monthly. The respondents were selected using a systematic sampling technique. In the systematic sampling technique, the selection of the respondents was based on a random starting point but with a predetermined, periodic interval. At the health facilities, on the days of the visit, the OPD registers were taken and estimated average OPD registrants (250) were divided by the estimated daily sample (32) to get the sampling interval. Following that, with the aid of a computer-assisted randomiser, the first participant was randomly selected/chosen, and the sampling interval was subsequently applied until the researchers had the required daily sample.

### Data collection tool/techniques

Questionnaire were developed for the data collection exercise (see supplementary file 1). Before data collection, the questionnaire were reviewed by experts to check validity and reliability of the data collection instrument. Additionally, the structured questionnaire was pretested among 30 patients at HART Adventist hospital, to check for validitiy, reliability, flow of information as well as questions that may seem sensitive for patients. After pretesting, the instrument was revised and some questions were rephrased or rewritten to enhance the reliability and validity of the data collection instrument. Additionally, before data collection, the finalised instrument was designed and saved electronically on an online KoboCollect server. The questionnaire was then administered using the computer-assisted Kobocollect software. The information/responses given by participants were entered on Android-based devices using the Kobocollect software, stored locally, and uploaded regularly to the Kobocollect online server. The data uploaded to the online server were managed by the researchers. During the data collection, respondents were approached individually by trained enumerators (not staff of the selected health facilities) and asked to participate in the study. The trained enumerators guided the consented clients in answering the research questions, and ensured their rights were not violated, coupled with helping them to choose appropriate venues to ensure privacy. The researchers upon completion of the data collection exercise, extracted the data for statistical analysis.

### Study variables

#### Dependent variable

The dependent variable in this study is whether patients were satisfied or dissatisfied with the service they received in these selected health facilities. The outcome variable satisfaction with health service was recoded into a binary variable with satisfied as “1” and dissatisfied as “0”.

#### Independent variables

The study considered 17 independent variables to help determine patient satisfaction with health service/delivery in the selected health facilities. The variables were age group, gender, educational level, religion, marital status, monthly income, insurance enrolment, the functionality of the facility, disability-friendly facilities, ventilated OPD, whether the facility has directional signs for navigation, and has adequate staff, description of the waiting area, environmental description, money spent on treatment, time spent at facility and attitude of health staff. These variables were selected because they have been used to predict patient satisfaction in Ghana and elsewhere [8, 25, 26].

### Data management and analytical procedures

Data for the study was downloaded from the online server upon completion of data collection. The data was then cleaned and managed using self-written commands. The data management and statistical analysis were performed using STATA statistical software version 16.0 [27]. Firstly, descriptive computations were conducted to describe the sampled general characteristics. Following that, at 95% confidence interval and 5% alpha level, two-level logistic models were built to determine the association between the dependent and independent variables. The first model (Model I) was the unadjusted model whilst Model II was the adjusted model that controlled for the effect of other covariates. The outputs of the regression model were reported in odds ratio and a corresponding 95% confidence interval. Collinearity diagnostics were performed before the multivariable regression and reported using the variance inflation factor (VIF), with a cut-off of 10. The results showed no evidence of collinearity between the independent variables (Mean VIF = 1.27, Maximum VIF = 1.61, Minimum VIF = 1.10) (See Supplementary file 2). Finally, the Hosmer-Lemeshow post-estimation test was utilized to assess the fitness of the model, and the results indicated no evidence of poor fit (See Table 3).

### Ethical consideration

In order to abide by the declaration of Helsinki, this study sought ethical clearance from the Committee on Human Research, Publication, and Ethics (CHRPE), the Institutional Review Board of the Kwame University of Science and Technology, with reference number (CHRPE/AP/619/19), which serves as ethical backing for the study. Moreover, permission was requested from the administrators of these selected health facilities and was granted. Before obtaining informed consent from patients, the study objectives, procedures and duration, and right to participation or withdrawal, including possible risks and benefits of the study were explained to participants. Informed consent was sought from respondents before data collection.

## Results

Table 2 presents the socio-demographic characteristics of the 385 patients who participated in the study. Nearly half of the participants (49.9%) visited Kumasi South Hospital, a little over one-third (34.6%) were in the age category of 30–39 years, and nearly one-third (31.7%) were between the ages of 18–29 years. The mean age was 36.0 (0.58) with an age range of 18–71 years. More than half of the participants (59.2%) were females, a little over half (51.4%) were married, and more than one-third (38.5%) had tertiary education. Majority of the participants (81.0%) were Christians and employed (83.9%). Majority of the participants (85.4%) were enrolled on the National Health Insurance Scheme (NHIS) and nearly two-thirds (60.3%) earn less than Ghana cedis (GHC) 1000 monthly.

**Table 2:** Socio-demographic characteristics of participants

Socio-demographic characteristics	Frequency (*n* = 385)	Percentage (100%)
**Health facilities**		
Cocoa clinic	95	24.7
Kumasi south hospital	192	49.9
Tafo hospital	98	25.4
**Age group [in years]**		
18–29	122	31.7
30–39	133	34.6
40–49	81	21.0
≥ 50	49	12.7
Mean ± (S.D)	36.02 ± 0.58	[18–71]
**Gender**		
Male	157	40.8
Female	228	59.2
**Marital status**		
Never married	142	36.9
Married	198	51.4
Separated/Divorced	20	5.2
Widowed	25	6.5
**Educational level**		
No formal education	29	7.5
Primary	30	7.8
JSS/JHS	54	14.0
SSS/SHS	124	32.2
Tertiary	148	38.5
**Religion**		
Christian	312	81.0
Moslem	67	17.4
Traditionalist	6	1.6
**Employment status**		
Employed	323	83.9
Unemployed	62	16.1
**Health insurance enrolment status**		
Uninsured	56	14.6
Insured	329	85.4
**Average monthly income (GHC)**		
< 1000	232	60.3
1000–2000	122	31.7
≥ 2001	31	8.0

**Fig. 2:**
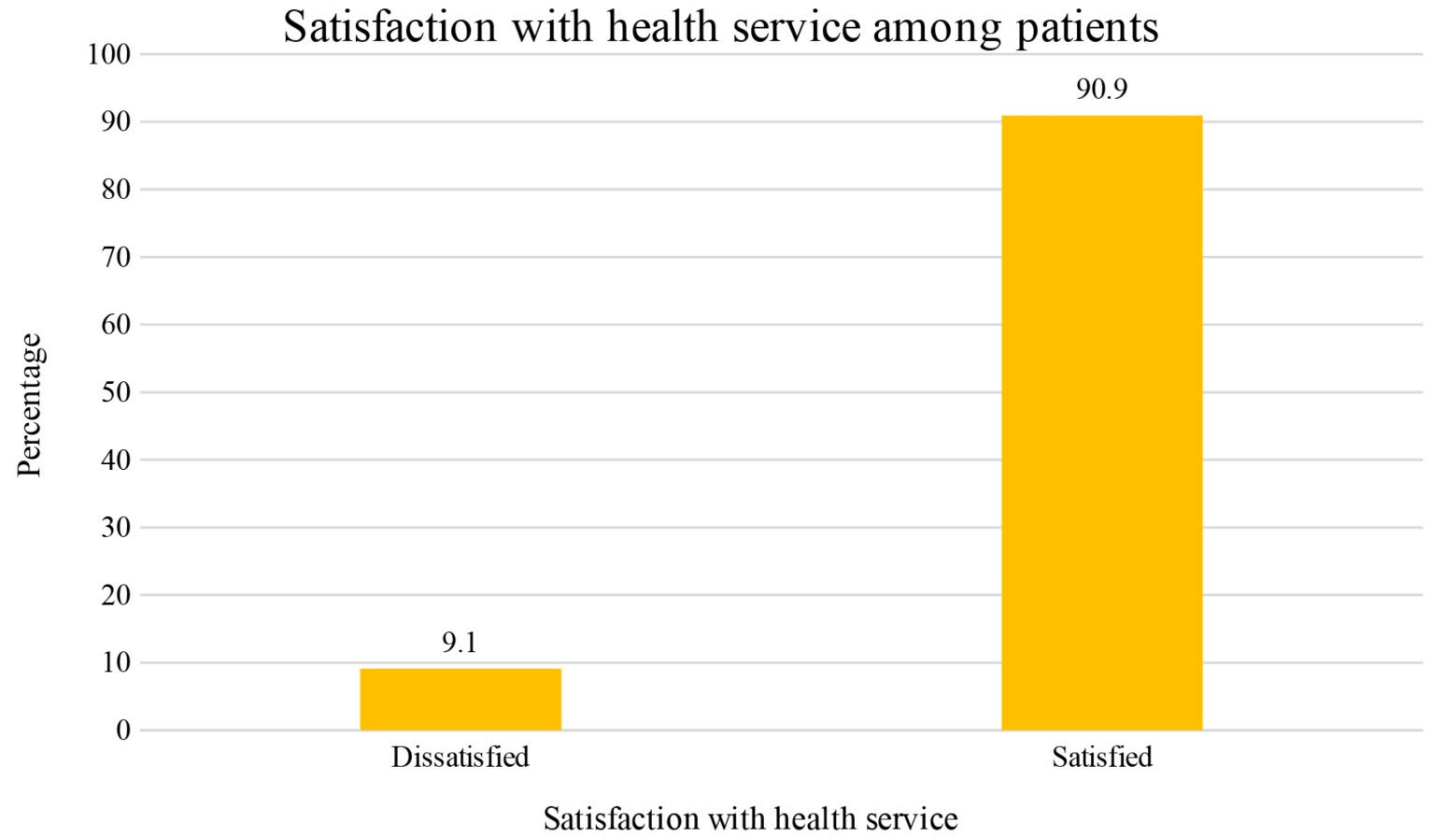
Satisfaction with health service among patients

Figure 2 is a pictorial presentation of the overall satisfaction with health services patients received at the outpatient departments of the selected health facilities. Almost all the participants (90.9%) were satisfied with the services they received whilst nearly a tenth (9.1%) were dissatisfied.

Table 3 presents a logistic regression result of the predictors of satisfaction with health services among patients. Participants who obtained JHS/JSS education had higher odds of satisfaction with service compared to those with no formal education [OR = 11.04, 95%CI = 1.22–19.70]. Respondents who were married compared to never married [OR = 3.29, 95%CI = 1.55–6.99], participants who agreed facility is disability-friendly compared to those who disagreed [OR = 11.00, 95%CI = 4.51–26.83], and participants who agreed OPD is ventilated compared to those who disagreed [OR = 14.42, 95%CI = 3.68–26.54] had higher odds of satisfaction with health service. Participants who agreed there are directional signs for navigation [OR = 4.15, 95%CI = 1.92–8.99], participants who agreed the facility has adequate staff [OR = 3.63, 95%CI = 1.01–13.80], and participants who agreed waiting area is comfortable and attractive [OR = 14.47, 95%CI = 9.20–25.05] had increased odds of satisfaction with health service. Participants who described the OPD environment as dirty [OR = 0.37, 95%CI = 0.14–0.98], those who spent more than 2 h at the health facility [OR = 0.11, 95%CI = 0.01–0.87], and participants who described staff attitude as irritating and rude [OR = 0.15, 95%CI = 0.06–0.35] had lower odds of satisfaction with health service.

**Table 3:** Predictors of satisfaction with health service/delivery

Variable	Unadjusted Model (Model I)		Adjusted Model (Model II)	
	OR	95% C. I	AOR	95%C. I
**Age group**				
18–29 (ref)	1		-	
30–39	2.13	[0.94–4.81]	-	-
40–49	2.63	[0.93–7.40]	-	-
≥ 50	4.07	[0.91–10.25]	-	-
**Gender**				
Male (ref)	1		-	
Female	0.97	[0.47–1.96]	-	-
**Educational level**				
No formal education (ref)	1		1	
Primary	1.86	[0.40–8.69]	0.27	[0.03–2.77]
JHS/JSS	11.04*	[1.22–19.70]	1.17	[0.08–7.40]
SHS	2.66	[0.82–8.65]	2.05	[0.30–7.96]
Tertiary	1.61	[0.54–4.77]	1.01	[0.16–6.48]
**Religion**				
Christian (ref)	1		-	
Moslem	0.64	[0.28–1.50]	-	-
**Marital status**				
Never married (ref)	1		1	
Married	3.29**	[1.55–6.99]	3.06*	[1.07–8.74]
Divorced/separated	0.84	[0.48–7.61]	0.92	[0.74–14.56]
Widowed	4.64	[0.60–16.02]	7.91	[0.62–10.28]
**Monthly income (GHC)**				
< 1000 (ref)	1		-	
1000–2000	0.86	[0.41–1.83]	-	-
≥ 2001	0.88	[0.25–3.15]	-	-
**Health insurance enrolment**				
Uninsured (ref)	1		-	
Insured	1.24	0.49–3.14]	-	-
**Functional facilities**				
All (ref)	1		-	
Some	0.58	[0.21–1.61]	-	-
**Disability friendly**				
No (ref)	1		1	
Yes	11.00**	[4.51–26.83]	7.93**	[2.07–14.43]
**Ventilated OPD**				
No (ref)	1		1	
Yes	14.42***	[3.68–26.54]	2.03	[0.20–10.67]
**Directional signs for navigation**				
Don’t know (ref)	1		1	
No	0.38	[0.09–1.56]	0.54	[0.05–6.35]
Yes	4.15***	[1.92–8.99]	3.12*	[1.92–10.59]
**Facility has adequate staff**				
Disagree (ref)	1		1	
Neutral	0.42	[0.10–1.78]	0.12	[0.02–1.07]
Agree	3.63*	[1.01–13.80]	0.70	[0.09–5.29]
**Waiting area comfortable and attractive**				
Disagree (ref)	1		1	
Neutral	3.03	[0.98–9.27]	2.54	[0.47–12.76]
Agree	14.47***	[9.20–25.05]	10.02**	[2.35–22.63]
**Money spent on treatment/care**				
< 100 (ref)	1		-	
100–300	1.04	0.47–2.28]	-	-
301–500	1.81	[0.68–4.86]	-	-
501	4.38	[0.56–14.	-	-
**Environmental description**				
Promotes privacy (ref)	1		1	
Noisy	0.57	[0.23–1.41]	0.97	[0.20–4.66]
Quiet	1.18	[0.37–3.79]	1.13	[0.21–5.99]
Clean	2.20	[0.48–10.06]	0.90	[0.11–7.20]
Dirty	0.37*	[0.14–0.98]	0.34	[0.07–1.64]
**Time spent at facility**				
< 30 min (ref)	1		1	
30–59 min	0.22	[0.03–1.81]	0.75	[0.07–7.70]
1–2 h	0.29	[0.04–2.42]	0.84	[0.08–9.49]
> 2 h	0.11*	[0.01–0.87]	0.45*	[0.04–0.93]
**Attitude of health staff**				
Kind and welcoming (ref)	1		1	
Respect and patient	1.04	[0.42–2.55]	0.60	[0.18–2.00]
Irritating and rude	0.15***	[0.06–0.35]	0.14**	[0.04–0.51]
**Goodness of fit-test**				
**Hosmer-Lemeshow test**		**X**^**2**^ **= 6.79**		**P-value = 0.560**

*Source* 95%CI = 95% confidence intervals in brackets, OR = Odds Ratio, AOR = Adjusted Odds Ratio, ^*^p < 0.05, ^**^p < 0.01, ^***^p < 0.001, 1 = Reference category

In the adjusted model (Model II), married respondents compared to never married [AOR = 3.06, 95%CI = 1.07–8.74] and participants who agreed facility is disability-friendly compared to those who disagreed [AOR = 7.93, 95%CI = 2.07–14.43] had higher odds of satisfaction with health service. Participants who agreed facility has directional signs for navigation compared to those who disagreed [AOR = 3.12, 95%=1.92–10.59] and participants who agreed the waiting area is comfortable and attractive compared to those who disagreed [AOR = 10.02, 95%CI = 2.35–22.63] had higher odds of satisfaction with health service. Participants who spent more than 2 h at a health facility compared to those who spent less than 30 min [AOR = 0.45, 95%CI = 0.04–0.93] and participants who described the health staff attitude as irritating and rude [AOR = 0.14, 95%CI = 0.04–0.51] had lower odds of satisfaction with health service.

## Discussion

Access to healthcare is an important indicator of both the progress and realization of universal health coverage. The study was conceptualized on the assumption that highly satisfied patients will access healthcare due to its perceived quality as operationally defined in this study. We investigated patients’ satisfaction with the health service they received and factors that influence satisfaction in selected facilities in Kumasi. The key findings were that almost all the participants (90.9%) were satisfied with the services they received, and that, satisfaction is influenced by varying factors. For instance, being married, and agreeing that facility is disability-friendly, and has directional signs for navigation, and waiting area is comfortable and attractive predicted satisfaction with health services. Moreover, spending more than 2 h at the health facility and having irritating and rude health staff were protective of satisfaction with health service.

Patients utilize health facilities with the expectation of receiving quality healthcare which will bring about satisfaction and general wellbeing. Hence, getting the subjective views of patients on quality care is essential and a realistic yardstick to help evaluate and improve the health services patients receive [28]. The study revealed that almost all the participants (90.9%) were satisfied with the health service provided to them. The finding is similar to a cross-sectional study conducted in Accra, Ghana where the authors found that more than two-thirds (69.5%) of the participants were satisfied with the service they received [19]. Also, in Sunyani Ghana, the overall satisfaction of patients concerning the service quality of the hospital was found to be good as nearly three-fourths (73.4%) were satisfied with the service they received [21]. In Ethiopia, a study showed overall patients’ satisfaction level of 89.3% among patients visiting outpatient departments of health facilities [29]. In 2018, Kulkarni affirmed in India that nearly three-fourths (73%) of the participants were satisfied with the services they were provided with at the OPDs [4]. Although, all these studies reported on patients’ satisfaction with health care, there are variations pertaining to satisfaction among patients. All the studies reported relatively lower satisfaction level compared to our study which could be influenced by differences in research methodologies, sample sizes, and study settings. Additionally, our study was conducted in an urbanized area, specifically the second most advanced city in Ghana, which features superior healthcare infrastructure, advanced diagnostic facilities, higher-tier hospitals, and specialized health professionals. These factors likely contributed to the higher overall satisfaction levels observed in our study.

The study revealed that respondents who were married compared to never married had higher odds of satisfaction with health services. In congruence with the research findings, a study conducted in Turkey revealed that married couples were satisfied with the health care or delivery they were provided with [30]. Being satisfied with the quality of care as a married individual would translate into increased healthcare utilization [31]. This finding is in line with a cross-sectional study conducted in Saudi Arabia where the authors revealed that the married couples attending tertiary facilities were satisfied with the health service or care received [32]. In congruence with the above finding, a study in Ghana also revealed that married participants were satisfied with the health services they were provided with [33]. This finding solidifies the evidence that marriage brings about social protection, and that this social network due to marital status helps to raise patient satisfaction [32]. The plausible explanation is that marriage brings about some sense of social dignity and respect and this could translate into health providers respecting and treating them with care leading to higher satisfaction [34, 35].

Provision of disability-friendly facilities has the tendency to heighten healthcare accessibility and achieve universal healthcare coverage [36]. This study revealed that participants who agreed facility is disability-friendly had higher odds of satisfaction with health service. In Bangladesh, a mixed-methods study revealed that health facilities accessed by clients were not disability-friendly leading to higher levels of dissatisfaction with service [37]. In relation to our findings, a study in the United States of America found that the unavailability of disability-friendly facilities coupled with payment conditions translated into elderly people with and without disability expressing dissatisfaction with health care [38]. Unavailability of disability-friendly facilities could posit that the health needs of people with disability and even the aged are excluded leading to dissatisfaction, whilst the availability of disability-friendly facilities means otherwise, leading to higher levels of satisfaction [39].

The study revealed that participants who agreed facility has directional signs for navigation and that the waiting area is comfortable and attractive had higher odds of satisfaction with health service. In tandem with the study’s findings, a study conducted in Saudi Arabia revealed that patients were satisfied with the service they received at the health facility if the facility had basic amenities, including comfortable waiting space [40]. As showed by Xuan et al., 2021, in their study in China, the availability of directional signs, visual arts, and comfortable waiting areas is linked to satisfaction among patients [41]. The plausible explanation for the finding is that the unavailability of directional signs, signage, visual arts, and uncomfortable waiting areas would result in patients being aggressive and stressed translating into poor satisfaction with health services [41]. This is because the availability of signage, directional signs, and visual arts would reduce stress and aggressiveness thereby improving patient satisfaction [42].

Regarding the accessibility of healthcare services at the regular OPDs and in the overall model of patient’s satisfaction, waiting time was a statistically significant variable [29]. The study revealed that participants who spent more than 2 h at a health facility had lower odds of being satisfied with health service. In a cross-sectional study conducted in Ethiopia, the authors reported that the patients who had waited 61–120 min had their satisfaction levels decreased by 78% and those who had waited 121–180 min by 87% [29]. In relation to the above finding, research conducted in Northwest Ethiopia revealed that long waiting time to see a doctor after registration was found to be negatively associated with the level of patient’s satisfaction [43]. The long waiting time as revealed in the study could be attributed to the loads of work/pressure coupled with limited health professionals at these facilities. Also, following the absence of online booking of appointments and payment methods, patients are mostly overcrowded at OPDs and waiting to be attended to by health professionals which translates into increased waiting time and subsequently dissatisfaction with service [44].

The study revealed that participants who described the health staff attitude as irritating and rude had lower odds of satisfaction with health service. Corroborating and affirming the study’s findings, a mixed-methods study conducted in Ghana revealed that the ill-practices and poor attitude of health professionals translated into dissatisfaction among health services patients received [45]. Consistent with the study’s findings, qualitative research conducted in the Eastern region of Ghana revealed that patients were satisfied with the care they received following the positive attitude of health staff [35]. Corroborating and affirming the study’s findings, a study in Nigeria revealed that health staff had poor attitudes towards patients coupled with abandoning and neglecting patients, fraudulently collecting money from patients and giving preferential treatment led to patient dissatisfaction with service [46]. Late and low salary payments, no promotion, inadequate health staff, and workload/pressure among others could lead to negative attitudes of health professionals which subsequently translate into patient’s dissatisfaction with health service [46].

## Strengths and limitations of the study

The study had some strengths and weaknesses positing that results should be interpreted with caution. The study’s strength resides in its novelty as the first study to investigate patient satisfaction with quality of care at the outpatient departments in Kumasi. Additionally, the use of appropriate methodology and rigorous analysis is another strength. Despite the strength of the study, it has weaknesses that cannot be overemphasized. The study was limited to the adoption of the cross-sectional study design making it impossible to ascertain causal inference. There might be social desirability flaws following the fact that the study was institutional-based and patients will tend to produce positive responses. Additionally, patient satisfaction was measured based on patient’s self-report which may introduce recall bias, and that patients reported what may not reflect the true satisfaction with health service. These weaknesses notwithstanding, the rigorous procedures adopted in our study were able to suppress the effects, thus making the findings viable for decision making.

## Conclusion

The study showed a high level of patient satisfaction with health services. Additionally, being married, and agreeing that the facility is disability-friendly, has directional signs for navigation, and the waiting area is comfortable and attractive predicted satisfaction with health services. The study findings call for the need to develop and implement health delivery interventions and strategies (i.e.patient-centered interventions, disability-friendly facilities, and sustainability and improvement of quality service) to improve and sustain patient satisfaction levels with health care service. These strategies must be directed towards addressing inequalities in infrastructural development and inputs needed for healthcare delivery in the health systems.

## Implications for practice and research

The findings of this study would assist health practitioners and policymakers in developing strategies and focusing on improving service delivery areas to help improve the satisfaction of clients. Future studies should be conducted to explore the acceptability and feasibility of online appointment booking and payment for public health facilities in Ghana in order to reduce OPD overcrowding and long waiting times. Future studies should also investigate the links between health staff strengths, motivation (intrinsic and extrinsic) and attitude towards patients.

## Electronic supplementary material

Below is the link to the electronic supplementary material.

Supplementary Material 1

Supplementary Material 2

## Data Availability

The datasets generated and/or analysed in this study are freely available upon making a reasonable request to the corresponding author.

## References

[1] Piabuo SM, Tieguhong JC. Health expenditure and economic growth - a review of the literature and an analysis between the economic community for Central African states (CEMAC) and selected African countries. Health Econ Rev. 2017;7(23):1–13. 28593509 10.1186/s13561-017-0159-1 PMC5462666

[2] Mosadeghrad A. A conceptual Framework for Quality of Care. Mater Socio Med. 2012;24(4):251. 10.5455/msm.2012.24.251-261 PMC3732361 23922534

[3] Haemmerli M, Powell-Jackson T, Goodman C, Thabrany H, Wiseman V. Poor quality for the poor? A study of inequalities in service readiness and provider knowledge in Indonesian primary health care facilities. Int J Equity Health [Internet]. 2021;20(1):1–12. 10.1186/s12939-021-01577-1 10.1186/s12939-021-01577-1 PMC8567576 34736459

[4] Kulkarni SK. A study of patient satisfaction level in Out Patient Department (OPD) in a tertiary care hospital in Maharashtra. IOSR J Dent Med Sci. 2018;17(3):31–9.

[5] World Health Organization Regional Office for Europe. Patient satisfaction and experience at migrant health centres in Turkey. World Heal Organ. 2021.

[6] Shahzad S, Hamid S, Masood Z, Mustafa R. Patient satisfaction; OPD services in a tertiary care hospital of Lahore. Prof Med J. 2013;20(6):973–80.

[7] Mohd SA, Chakravarty BA. Patient satisfaction with services of the outpatient department. Med J Armed Forces India. 2014;70:237–42. 25378776 10.1016/j.mjafi.2013.06.010 PMC4213903

[8] Amporfro DA, Boah M, Yingqi S, Cheteu Wabo TM, Zhao M, Ngo Nkondjock VR, et al. Patients satisfaction with healthcare delivery in Ghana. BMC Health Serv Res. 2021;21(1):1–13. 34294102 10.1186/s12913-021-06717-5 PMC8299658

[9] Dalaba MA, Welaga P, Matsubara C. Cost of delivering health care services at primary health facilities in Ghana. BMC Health Serv Res. 2017;17(1):1–11. 29149853 10.1186/s12913-017-2676-3 PMC5693519

[10] Marrone P. Health and healthcare in Ghana, 1957–2017. Etica E Polit. 2013;15(1):583–605.

[11] Mensah J, Asamoah D, Tawiah AA. Optimizing patient Flow and Resource utilization in Out Patient Clinic: a comparative study of Nkawie Government Hospital and Aniwaa Health Center. J Appl Bus Econ. 2014;16(3):181–8.

[12] Tenkorang EY. Health Provider Characteristics and Choice of Health Care Facility among Ghanaian Health Seekers Health Provider Characteristics and Choice of Health Care Facility among Ghanaian Health seekers. Heal Syst Reform. 2016;8604. 10.1080/23288604.2016.1171282 31514639

[13] Bamfo BA, Dogbe CSK. Factors influencing the choice of private and public hospitals: empirical evidence from Ghana. Int J Pharm Healthc Mark. 2017;11(1):80–96. 10.1108/IJPHM-11-2015-0054

[14] Amponsah E. Patient’s preference of public and private hospitals: Evidence from Kumasi and Bibiani in the Ashanti and Western regions of Ghana. 2015;11(1):1–15. 10.1108/IJPHM-11-2015-0054

[15] Das J, Woskie L, Rajbhandari R, Abbasi K, Jha A. Rethinking assumptions about delivery of healthcare: implications for universal health coverage. BMJ. 2018;361. 10.1136/bmj.k1716 PMC5961312 29784870

[16] Escribano-Ferrer B, Cluzeau F, Cutler D, Akufo C, Chalkidou K. Quality of Health Care in Ghana: Mapping of interventions and the Way Forward. Ghana Med J. 2016;50(4):238–47. 28579630 10.4314/gmj.v50i4.7 PMC5443676

[17] Kidanemariam G, Gebrekidan H, Dagnazgi EA, Asfaw K. Improving patient satisfaction and Associated Factors at Outpatient Department in General hospitals of Central Zone, Tigray, Northern Ethiopia, June 2018-August 2019 : pre- and Postinterventional Study. Biomed Res Int. 2023;2023:1–8. 10.1155/2023/6685598 PMC10665098 38027041

[18] Essiam JO. Service quality and patients satisfaction with Healthcare Delivery: empirical evidence from patients of the Out Patient Department of a Public University Hospital in Ghana. Eur J Bus Manag. 2013;5(28).

[19] Odonkor ST, Frimpong C, Duncan E, Odonkor C. Trends in patients ’ overall satisfaction with healthcare delivery in Accra, Ghana. Afr J Prim Care Fam Med. 2019;1–6. 10.4102/phcfm.v11i1.1884 PMC6779968 31588770

[20] Ahenkan A, Aduo-Adjei K. Predictors of patient satisfaction with quality of Healthcare in University hospitals in Ghana. Hosp Pract Res. 2017;2(1):9–14. 10.15171/hpr.2017.03

[21] Peprah AA, Atarah BA. Assessing Patient ’ s Satisfaction Using SERVQUAL Model: A Case of Sunyani Regional. 2014;(February):133–43.

[22] Ghana Statistical Service (GSS). Ghana 2021 Population and Housing Census. 2021;3A.

[23] Charan J, Biswas T. How to calculate sample size for different study designs in medical research? Indian J Psychol Med. 2013;35(2):121–6. 24049221 10.4103/0253-7176.116232 PMC3775042

[24] Shure G, Gamachu M, Mitiku H, Deressa A, Eyeberu A, Mohammed F, et al. Patient satisfaction and associated factors among insured and uninsured patients in Deder General Hospital, eastern Ethiopia: a facility-based comparative cross-sectional study. Front Med. 2023;10(December):1–11. 10.3389/fmed.2023.1259840 PMC10777387 38204483

[25] Ferreira DC, Vieira I, Pedro MI, Caldas P, Varela M. Patient satisfaction with Healthcare Services and the techniques used for its Assessment: a Systematic Literature Review and a bibliometric analysis. Healthc. 2023;11(5). 10.3390/healthcare11050639 PMC10001171 36900644

[26] Babatola OH, Popoola RO, Olatubi MI, Adewoyin FR. Patients’ satisfaction with Health Care Services in Selected Secondary Health Care Facilities in Ondo State, Nigeria. J Fam Med Dis Prev. 2022;8(1):1–9. 10.23937/2469-5793/1510145

[27] StataCorp. Stata Statistical Software: release 16. College Station. TX: StataCorp LLC; 2021. pp. 2021–3.

[28] Michie S, Yardley L, West R, Greaves F. Developing and Evaluating Digital Interventions to Promote Behavior Change in Health and Health Care: Recommendations Resulting From an International Workshop Corresponding Author : J Med INTERNET Res. 2017;19(6):1–13. 10.2196/jmir.7126 PMC5509948 28663162

[29] Geberu DM, Biks GA, Gebremedhin T, Mekonnen TH. Factors of patient satisfaction in adult outpatient departments of private wing and regular services in public hospitals of Addis Ababa, Ethiopia : a comparative cross- sectional study. BMC Health Serv Res. 2019;19:1–13. 31752821 10.1186/s12913-019-4685-x PMC6873435

[30] Ayranci E, Atalay N. Demographic determinants of patient satisfaction: a study in a Turkish context. Int J Acad Res Bus Soc Sci. 2019;9(6):837–47.

[31] Pandey KR, Yang F, Cagney KA, Smieliauskas F, Meltzer DO, Ruhnke GW. The impact of marital status on health care utilization among Medicare bene fi ciaries. Med (Baltim). 2019;98(12):1–8. 10.1097/MD.0000000000014871 PMC6709281 30896632

[32] Aljarallah NA, Almuqbil M, Alshehri S, Mohammed A, Khormi S, Alreshaidan RM et al. Satisfaction of patients with health care services in tertiary care facilities of Riyadh, Saudi Arabia: a cross-sectional approach. Front Public Heal. 2023;1–11. 10.3389/fpubh.2022.1077147 PMC9880422 36711344

[33] Amu H, Nyarko SH. Satisfaction with Maternal Healthcare Services in the Ketu South Municipality, Ghana : A Qualitative Case Study. 2019;2019. 10.1155/2019/2516469 PMC6481155 31093496

[34] Baloyi GT. Marriage and Culture within the Context of African Indigenous Societies: A Need for African Cultural Hermeneutics. 2022;48(1):1–12.

[35] Diema K, Afram J, Doat A, Mensima R, Asibi J, Mohammed I et al. Influence of nurse-patient relationship on hospital attendance. A qualitative study of patients in the Kwahu Government Hospital, Ghana. Heliyon [Internet]. 2021;7(2):e06319. 10.1016/j.heliyon.2021.e06319 10.1016/j.heliyon.2021.e06319 PMC7907811 33665463

[36] Izzati N, Ghani A, Ismail K, Salleh NA. Provis Disabl Facilities Public Hosp. 2016;00081:1–8.

[37] Torsha N, Rahman FN, Hossain S, Chowdhury HA, Kim M, Rahman SMM et al. Disability – friendly healthcare at public health facilities in Bangladesh: a mixed – method study to explore the existing situation. BMC Health Serv Res [Internet]. 2022;1–12. 10.1186/s12913-022-08538-6 10.1186/s12913-022-08538-6 PMC9490997 36127659

[38] Iezzoni LI, Davis RB, Soukup J, Day BO. Satisfaction with quality and access to health care among people with disabling conditions. Int J Equity Health. 2002;14(5):369–81. 10.1093/intqhc/14.5.369 12389803

[39] Magnusson L, Kebbie I, Jerwanska V. Access to health and rehabilitation services for persons with disabilities in Sierra Leone – focus group discussions with stakeholders. BMC Health Serv Res [Internet]. 2022;1–11. 10.1186/s12913-022-08366-8 10.1186/s12913-022-08366-8 PMC9356469 35932077

[40] Aldebasi YH, Ahmed MI. Patients ’ satisfaction with Medical services in the Qassim Area. Heal Manag Policy Sect. 2011;5(4):813–7.

[41] Xuan X, Arch M, Ap L, Li Z, Arch M, Chen X, et al. Study of the Physical Environment of Waiting Areas and its effects on patient satisfaction, experience, Perceived Waiting Time, and Behavior in China. Cent Heal Des. 2021;485:1–16. 10.1177/1937586721989058 33511886

[42] Maqbool T, Raju S, In E. Importance of patient-centred signage and navigation guide in an orthopaedic and plastics clinic. BMJ Qual Improv Rep. 2016;1–5. 10.1136/bmjquality.u209473.w3887 PMC4752701 26893888

[43] Eshetie G, Feleke A, Genetu M. Patient satisfaction and Associated Factors among Outpatient Health Service users at primary hospitals of North Gondar, Northwest Ethiopia, 2016. Adv Public Heal. 2020;2020:1–8. 10.1155/2020/6102938

[44] Zhang H, Ma W, Zhu J, Wang L, Guo Z, Chen X. How to adjust the expected waiting time to improve patient ’ s satisfaction ? BMC Health Serv Res. 2023;23(455):1–8. 37158912 10.1186/s12913-023-09385-9 PMC10169334

[45] Amoah PA, Nyamekye KA, Addo EO. A multidimensional study of public satisfaction with the healthcare system: a mixed-method inquiry in Ghana. BMC Health Serv Res [Internet]. 2021;1–17. 10.1186/s12913-021-07288-1 10.1186/s12913-021-07288-1 PMC8656047 34886857

[46] Isaruk ID, Isaruk JID, George DT. Attitude and Ethical Behaviors of Healthcare Providers as Antidotes of Health Service Consumer Satisfaction in Mgbuoshimini Primary Health Centre, Port Harcourt, Nigeria. J Heal Appl Sci Manag. 2023;6(3):26–31.

