# Supplementary material for: Patient satisfaction with quality of care at out-patient departments in selected health facilities in Kumasi, Ghana
Source: BMC Health Serv Res. 2024 Sep 4;24:1027. doi: 10.1186/s12913-024-11399-w (PMC11375930; doi:10.1186/s12913-024-11399-w)
Supplement: Supplementary file 1 — Supplementary Material 1 [file 12913_2024_11399_MOESM1_ESM.docx]

# APPENDIX 1: QUESTIONNAIRE FOR CLIENTS

**KWAME NKRUMAH UNIVERSITY OF SCIENCE AND TECHNOLOGY/COLLEGE OF HEALTH SCIENCES/SCHOOL OF PUBLIC HEALTH/ DEPARTMENT OF HEALTH POLICY, MANAGEMENT AND ECONOMICS, KUMASI**

***Research Title:* *Patient satisfaction with quality of care at OPDs of selected health facilities***

**Introduction**

*Good morning/afternoon. I am a student at School of Public Health, KNUST. I will be conducting several meetings with people like you in selected hospitals in Kumasi to* *assess public perception of health service quality at various OPDs****.*** *Your opinions are highly essential at the same time vital as they will help us to improve the kind of service we provide.* *Whatever you say will be treated confidential, so feel at ease to express your candid opinion****.*** *Be assured that your responses will not in any way be linked to your identity*. *You are kindly requested to answer the questions below by indicating a tick or writing the appropriate answer when needed.*

***THANK YOU***

**Questionnaire number:**

**Date of Interview:**

**Interviewer Name:**

**SECTION A: Socio-demographic Data**

1. Health Facility: a. Kumasi South [ ] b. Cocoa Clinic [ ] c. Tafo Hospital [ ]

2. Age (years): …………

3. Marital status: a. Single [ ] b. Widowed [ ] c. Married [ ] d. Separated [ ] e. Divorced [ ]

4. Education level: a. Primary [ ] b. JHS [ ] c. SHS [ ] d. Tertiary [ ] e. None [ ]

5. Religion: a. Christian [ ] b.Muslim [ ] c.Traditional [ ] d. Others [ ]

6. Occupation: ………………………………………………………………………

7. Health insurance: a. Yes [ ] b. No [ ]

8. Average income per month (GHS): …………………………………………………………

**SECTION B: Structure of Health Care Provider** **Infrastructure**

8. What type of facilities are available at the health facility? a. Consulting Room [ ] b. Pharmacy [ ] c. Laboratory [ ] d. Washrooms [ ]

e. Radiology department [ ] f. Other ………………………………………….

9. How many of these facilities present in the health facility are functional?

a. All [ ] b. Some [ ] c. None [ ]

10. Are the infrastructures within the health facility disability friendly?

a. Yes [ ] b. No [ ]

11. How many exits are available at the OPD? ……………………..

12. How many fire extinguishers are at the OPD?..............................

13. Is the OPD well ventilated? a. Yes [ ] b. No [ ]

**Environment**

14. What best describes the environment of the health facility? Tick as many as applicable

a. Promotes privacy [ ] b. Noisy [ ] c. Quiet [ ] d. Clean [ ] e. Dirty [ ]

15. Are the directional signs to help with easy navigation around the OPD?

a. Yes [ ] b. No [ ] c. I don’t know [ ]

**Equipment**

16. What kind of equipment is used at the OPD? Tick as many as applicable

a. Thermometer [ ] b. Weighing scale [ ] c. Sphygmomanometer [ ] d. Glucometer [ ]

e. Stethoscope [ ] f. Computer [ ]

17. In your opinion which of these is inadequate in terms of quantity?

a. Thermometer [ ] b. Weighing scale [ ] c. Sphygmomanometer [ ] d. Glucometer [ ]

e. Stethoscope [ ] f. Computer [ ]

18. In your opinion which of these is inadequate in terms of quality?

a. Thermometer [ ] b. Weighing scale [ ] c. Sphygmomanometer [ ] d. Glucometer [ ]

e. Stethoscope [ ] f. Computer [ ]

**SECTION C: Processes and Activities at the OPD**

19. How much time does it take for you to be attended to by a health professional when you get to the OPD? …………………………..(mins)

20. How much time do you usually spend at the health practitioners office? ……………………(mins

21. On average, how much time do you spend at the hospital (from entry to exit)?............(hrs)

22. What attitude does the staff at the OPD usually show toward you? Tick as many as applicable.

a. Kind [ ] b. Welcoming [ ] c. Respectful [ ] d. Patient [ ] e. Compassionate [ ] f. Irritable [ ]

g. Rude [ ] h. Inpatient [ ] i. In-sensitive to my needs [ ]

**SECTION D. Outcome of Health Delivery**

23. What is your expectation when you visit the health facility? Circle as many as apply

a. To be Healed

b. To obtain all medications prescribed

c. To be attended to on time

d. To understand my medical condition

e. To be able to do all the requested investigations at the facility

Other…………………………………………..

24. Which of the expectations in Q 23 were met? Circle as many as apply

a. To be Healed

b. To obtain all medications prescribed

c. To be attended to on time

d. To understand my medical condition

e. To be able to do all the requested investigations at the facility

25. On average how much money do you spend at the hospital?

a. less than 100ghc [ ] b. 100 – 300ghc [ ] c. 300 - 500gh [ ] d. 500 - 700ghc [ ]

e. 700-900ghc [ ] f. more than 900ghc [ ]

26. Are you highly satisfied with the healthcare services at the OPD?

a. Yes [ ] b. No [ ]

**SECTION E: Quality of Healthcare Rating**

**In this section, I would like you to rate the overall quality of health care at the OPD. Based on your experiences with the health facilities, rate each quality parameter by ticking (√) one answer from the Likert scale where 1-Strongly disagree, 2-Disagree, 3-Neutral, 4-Agree, 5-Strongly Agree**

| **Quality parameter** | **1** | **2** | **3** | **4** | **5** |
| --- | --- | --- | --- | --- | --- |
| **Infrastructure, Environment and Equipment** | | | | | |
| 8. The waiting area at the OPD is comfortable and visually attractive |  |  |  |  |  |
| 9. The OPD is clean and tidy |  |  |  |  |  |
| 10. The OPD has clean washrooms for patients and visitors |  |  |  |  |  |
| 11. The OPD structures are disability friendly |  |  |  |  |  |
| 12. Equipment available at the OPD are modern |  |  |  |  |  |
| 13. There are directional signs to help with easy navigation |  |  |  |  |  |
| 14. The facility has a dispensary for OPD clients |  |  |  |  |  |
| 15. The facility has laboratory services available |  |  |  |  |  |
| **Workforce** | | | | | |
| 16. The facility has enough health care providers stationed at the OPD |  |  |  |  |  |
| 17. The health workers at the OPD create a friendly atmosphere for patients to feel safe and relaxed |  |  |  |  |  |
| 18. Health practitioners at the OPD are professional and competent |  |  |  |  |  |
| 19. Health workers at the OPD take time to listen to patient complaints |  |  |  |  |  |
| 20. Health workers at the OPD are compassionate |  |  |  |  |  |
| 21. Health workers dress professionally | | | | | |
| **Processes of the Health Care Provider** | | | | | |
| 22. Patients wait for a short time before they see a doctor |  |  |  |  |  |
| 23. The doctor attends to one patient at a time |  |  |  |  |  |
| 24. Patients are thoroughly examined by the healthcare provider |  |  |  |  |  |
| 25. Patients wait for a long time to get their medications |  |  |  |  |  |
| 26.Tests conducted at the OPD are safe |  |  |  |  |  |
| 27. Patients do not incur unnecessary cost at the OPD |  |  |  |  |  |
| 28. The charge for services at the OPD is affordable |  |  |  |  |  |
| 29. I was given adequate information on my health condition |  |  |  |  |  |
| 30. I was given adequate information on my treatment and its side effects |  |  |  |  |  |
| 31. I am relieved of my complaints after following the doctor’s instructions |  |  |  |  |  |
| 32. I am highly satisfied with the services provided at the OPD |  |  |  |  |  |

*Thank you for your cooperation*.
