# Supplementary material for: Patient satisfaction with quality of care at out-patient departments in selected health facilities in Kumasi, Ghana
Source: BMC Health Serv Res. 2024 Sep 4;24:1027. doi: 10.1186/s12913-024-11399-w (PMC11375930; doi:10.1186/s12913-024-11399-w)
Supplement: Supplementary file 2 — Supplementary Material 2 [file 12913_2024_11399_MOESM2_ESM.docx]

Appendix 2: Multicollinearity test results

| **Variable** | **VIF** | **Tolerance** | **R-Squared** |
| --- | --- | --- | --- |
| Educational level | 1.29 | 0.775 | 0.225 |
| Marital status | 1.10 | 0.909 | 0.091 |
| Disability friendly | 1.31 | 0.763 | 0.237 |
| OPD ventilation | 1.28 | 0.781 | 0.219 |
| Directional signs for navigation | 1.42 | 0.704 | 0.296 |
| Staff adequacy | 1.11 | 0.901 | 0.099 |
| Waiting area comfortable/attractive | 1.61 | 0.621 | 0.379 |
| Description of environment | 1.17 | 0.855 | 0.145 |
| Time spent at facility | 1.21 | 0.826 | 0.174 |
| Staff attitude | 1.15 | 0.870 | 0.130 |
| **Mean VIF** | **1.27** | |  |
